# Subclinical naming errors in mild cognitive impairment: A semantic deficit?

**DOI:** 10.1590/S1980-57642009DN20300010

**Published:** 2008

**Authors:** Indra F. Willers, Mónica L. Feldman, Ricardo F. Allegri

**Affiliations:** 1 Utrecht University, the Netherlands.; 2 Department of Neuropsychology (SIREN), CEMIC School of Medicine & Research Institute, Buenos Aires, Argentina.; 3 CONICET.

**Keywords:** amnestic, mild cognitive impairment, dementia, Alzheimer, naming

## Abstract

Mild cognitive impairment (MCI) is the transitional stage between normal aging and Alzheimer’s disease (AD). Impairments in semantic memory have been demonstrated to be a critical factor in early AD. The Boston Naming Test (BNT) is a straightforward method of examining semantic or visuo-perceptual processing and therefore represents a potential diagnostic tool. The objective of this study was to examine naming ability and identify error types in patients with amnestic mild cognitive impairment (aMCI).

**Methods:**

Twenty aMCI patients, twenty AD patients and twenty-one normal controls, matched by age, sex and education level were evaluated. As part of a further neuropsychological evaluation, all subjects performed the BNT. A comprehensive classification of error types was devised in order to compare performance and ascertain semantic or perceptual origin of errors.

**Results:**

AD patients obtained significantly lower total scores on the BNT than aMCI patients and controls. aMCI patients did not obtain significant differences in total scores, but showed significantly higher semantic errors compared to controls.

**Conclusion:**

This study reveals that semantic processing is impaired during confrontation naming in aMCI.

## Abstract (pt)

Comprometimento cognitivo leve (CCL) é um estágio de transição entre o envelhecimento normal e a doença de Alzheimer (DA). Comprometimento da memória semântica tem sido demonstrado como um fator crítico na DA precoce. O Teste de Nomeação de Boston (TNB) é um meio fácil para examinar o processamento semântico e viso-espacial e também um instrumento potencial de diagnóstico.

**Objetivo:**

Examinar a habilidade de nomeação e discriminar os tipos de erros em pacientes com CCL amnéstico (CCLa).

**Métodos:**

Vinte pacientes com CCLa, 20 pacientes com DA e 21 controles normais pareados por idade, sexo e nível educacional foram avaliados. Como parte da avaliação neuropsicológica a todos foi administrado o TNB. Uma ampla classificação dos tipos de erros foi realizada a fim de comparar o desempenho e conhecer a origem semântica ou perceptiva dos erros.

**Resultados:**

Os pacientes com DA obtiveram piores escores totais no TNB do que os pacientes com CCLa e controles. Os pacientes com CCLa não tiveram diferenças significativas nos escores totais, porém, mostraram um número maior e significativo de erros semânticos comparados aos controles.

**Conclusão:**

Este estudo revela que o processamento semântico está comprometido durante a nomeação por confrontação no CCLa.

Alzheimer’s disease (AD) is a major public health problem because of its growing prevalence and economic burden.^1^ An understanding of the prodromal states or early clinical presentations of AD is a significant priority since it would aide early detection, facilitate early treatment, and lead to prevention. There is a clinical cognitive continuum from normal aging through to AD. Cognitive decline without dementia has been commonly considered to be a normal consequence of brain aging, but can also indicate the onset of dementia. The boundary between normal aging and very early AD has become a central focus of research. The pre-dementia diagnosis is intimately connected to the development of therapies for the prevention of AD. This challenge explains the popularity of the concept of mild cognitive impairment (MCI) and its wide application in the epidemiological, clinical, paraclinical and therapeutic domains.^2^ In 1999, Petersen proposed a clinical continuum from normal aging through mild cognitive impairment to Alzheimer’s disease. Mild cognitive impairment was originally defined as the transitional state that can precede dementia.^3^ Mild cognitive impairment applies to individuals who have some degree of cognitive impairment but are not sufficiently debilitated as to warrant the diagnosis of dementia or AD. An individual with MCI typically develops memory deficit and soon exhibits other cognitive abnormalities without functional impairment.^3^

The original diagnostic criteria for mild cognitive impairment^3^ were:

- Memory complaint, preferably corroborated by an informant.
- Memory impairment relative to age-matched and education-matched healthy individuals.
- Preserved general cognitive function.
- Intact activities of daily living.
- Not clinically demented.

De Kosky and Chertkow (2001) proposed 3 subtypes of MCI: amnestic MCI (which is said to often evolve to Alzheimer’s disease), multiple domain MCI (which may represent normal aging or may progress to vascular cognitive impairment or neurodegenerative disorder), and single domain non-amnestic MCI (which may progress to fronto-temporal dementia, Lewy Bodies Dementia or Alzheimer Disease).^4^

In clinical-based studies the typical rate at which MCI patients’ progress to AD is 10 to 15% per year, which contrasts with incidence rates of the development of dementia in normal elderly subjects – 1–2% per year.^3^

Semantic memory, which refers to the general store of conceptual and factual knowledge, is a declarative and explicit memory system and a subcategory of long term memory.^5^ Previous studies have shown semantic memory as a major factor in neurological syndromes, such as language deficits and word finding problems (anomia) in AD, in addition to forms of aphasia and visual associative agnosia. Anomia is a frequent finding in very early AD.^6^ Performance on language batteries is one of several ways to examine semantic processing, such as verbal fluency,^7^ vocabulary testing on WAIS^8^ and the Boston Naming Test (BNT)^9^ for confrontation naming errors. These errors are characteristic of semantic deficits in particular and therefore represent a potential diagnostic tool.^10^ Semantic memory in MCI is under-investigated and some studies are controversial concerning impairment.^10-13^

The BNT is a visual confrontation naming test which consists of 60 schematic pictures of objects. Not only has the overall number of picture-naming errors been found to be related to global dementia severity, but also the analysis of AD-related increases in picture-naming errors has produced a rich set of data on specific cognitive-processing declines in AD. Previous studies show perceptual difficulties during confrontation naming as well as an increased number of visual perception errors, impaired phonological access and semantic representation.^14^ Balthazar et al. 2007 found no differences in total BNT scores in aMCI.^13^

The objectives of the present research were to study naming performance in aMCI and to compare the patterns of errors (visual or semantic) with normal controls and AD.

## Methods

### Participants

Twenty patients with aMCI (according to Petersen criteria, 1999^3^ and De Kosky and Chertkow criteria^4^), twenty patients with probable AD (NINCDS-ADRDA criteria according to McKhann et al.^16^) and twenty-one normal controls (NC) matched by age, sex and educational level were studied. Subjects were recruited from the Department of Neuropsychology (SIREN), CEMIC School of Medicine & Research Institute, Buenos Aires, Argentina and the Memory Centre from Hospital A. Zubizarreta, Government of Buenos Aires City, Argentina. All patients underwent extensive neurological, neuropsychological, neuropsychiatric, laboratory and neuroimaging assessments. Normal controls (NC) recruited from the general population comprised subjects without history of findings suggestive of neurological or psychiatric disease and who showed no evidence of cognitive impairment.

Patient demographic information is provided in Table 1.

**Table 1:** Demographic information.

	NC	aMCI	AD	p (ANOVA)
N	21	20	20	
Age (years)	72.6 (8.3)	74.1 (7.8)	74.4 (7.7)	<0.731
Sex (F/M)	10/10	12/8	9/11	ns*
Educational level (years)	12.8 (3.2)	15.0 (3.3)	13.5 (4.0)	<0.215
MMSE	28.6 (1.0)	28.3 (1.4)	17.6 (6.4)	NC vs aMCI=ns NC vs AD p<0.001

NC, normal controls; aMCI, amnestic mild cognitive impairment; AD, Alzheimer disease; MMSE, Mini Mental State Exam; Age, education and MMSE are expressed as mean (SD); Sex as number;

* from Chi square.

### Procedures

The study was performed according to CEMIC University’s institutional review board regulations and each participant gave oral informed consent. All subjects underwent a neuropsychological evaluation which consisted of the Mini Mental State Examination (MMSE),^17^ Signoret memory battery scale,^18^ trail making test A and B,^19^ semantic and phonologic verbal fluency,^20^ Wechsler abbreviated scale of intelligence,^21^ and Hamilton depression scale.^22^

The Spanish version of the Boston Naming Test (BNT) adapted in Buenos Aires by Allegri et al.^23^ was used for the study of naming.

Standard BNT administration^9^ was modified^23^ so that each subject began with item one and completed all 60 picture items. If a subject’s response indicated that a picture was misperceived, the examiner gave the appropriate semantic cue (error type 6) as per standard protocol. If a subject failed to name the item correctly within 20 sec, a standard phonemic cue was provided (error type 7). If a subject spontaneously self-corrected an error within 20 sec, full credit was given. These procedure was used to obtain scores of correct items and errors

In addition to investigating the accuracy and latency with which the pictures were named, the qualitative analysis or the type of errors that the groups made was also important. Errors were classified according to taxonomy of Hodges et al.^24^ and Lethlean and Murdoch^25^ as follows:

1. Semantically related errors
  - Semantic paraphasia: the given answer corresponds to a co-ordinate, super-ordinate or sub-ordinate category (e.g. ‘pen’ instead of ‘pencil’).
  - Adequate circumlocution: an adequate definition of the target, indirectly expressed through (several) other words (e.g. ‘something to measure temperature’ instead of ‘thermometer’).
  - Unclear circumlocution: an inadequate definition of the picture.
2. *Visuoperceptual errors visual* similarity:a misinterpretation of the intention of the picture (‘radio’ instead of ‘pencil sharpener’).
  - A part of the stimuli wrongly integrated: a misinterpretation of a single part of the picture (e.g. ‘fan’ instead of ‘helicopter’).
3. *Phonological errors:* phonemic paraphasias, the answer given is phonologically incorrect (e.g. ‘ballet’ instead of ‘palette’).
4. *Lack of answer:* when no answer was given, or when the subject did not know the name of the object or its use.
5. *Errors without any relation to* the stimuli
6. *Semantic anomia:* when a semantic cue was needed to form the right answer.
7. *Evocative anomia:* when a phonemic cue was needed to form the right answer.

### Statistical analysis

All analyses were carried out using SPSS version 13.0 (SPSS INC, Chicago, USA). For group comparison a descriptive analysis, an analysis of variance (ANOVA) and the Bonferroni test were used. For categorical variables and % chi square was used. The mean difference was considered significant at the p<0.05level.

## Results

The results showed a significant between-group difference in the total errors made during visual confrontation naming (p<0.000) for AD compared to aMCI and NC. The difference between aMCI and NC on total errors was not significant (p<0.119) (Table 2).

**Table 2:** Boston Naming Test: breakdown by error type.

Errors	NC	aMCI	AD	p
Total	6.52 (3.8)	11.1 (5.9)	27.3 (9.9)	0.000* 0.000** ns***
Semantic	1.0 (0.9)	6.3 (3.7)	11.4 (5.0)	0.000* 0.000** 0.000***
Visual perceptual	0.3 (0.6)	0.9 (1.2)	1.8 (1.7)	ns* 0.001** ns***
Phonological	0.0 (0.0)	0.2 (0.4)	0.1 (0.3)	ns* ns** ns***
Lack of answer	2.1 (1.1)	1.8 (1.7)	8.0 (6.6)	0.000* 0.000** ns***
Answer without relation	0.0 (0.0)	0.5 (0.8)	1.2 (2.5)	ns* 0.043** ns**
Cue provided Semantic cue	1.8 (1.9)	0.0 (0.0)	0.6 (0.9)	ns* 0.009** 0.000***
Phonemic cue	1.4 (0.9)	1.6 (1.3)	4.3 (4.0)	0.002* 0.001** ns***

NC, normal controls; aMCI amnestic mild cognitive impairment; AD, Alzheimer disease; MMSE, Mini Mental State Exam; Age, education and MMSE are expressed as mean (SD); Sex as number;

* AD vs aMCI;

** AD vs NC;

*** aMCI vs NC; p by multiple Bonferroni test, significant at the 0.05 level.

Analysis by type of error yielded a significant difference between aMCI patients and NC for semantic errors (p<0.000). No significant visual perceptual difference was found. aMCI patients had significantly less errors as regards lack of answer than AD patients (p<0.000) (Table 2).

**Figure 1:**
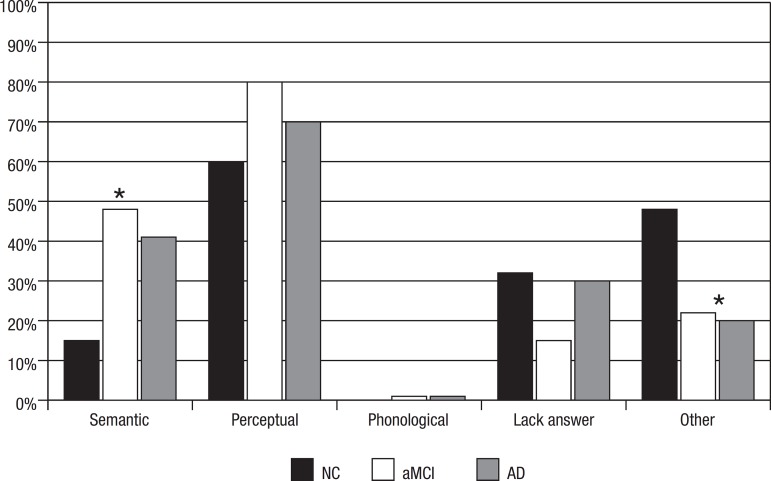
Analysis of naming error type (percentage) in NC, aMCI and AD. NC, normal controls; aMCI amnestic mild cognitive impairment; AD, Alzheimer disease. *p<.001.

Relative distribution of error type was also analyzed (Figure 1). Error distribution among MCI and AD patients was similar. The NC group made less (p<0.001) semantic errors (48% vs. 41% vs. 15% respectively) and more other errors than the MCI and AD groups, including answers without relation to the stimuli, semantic cue and phonemic cue (20% vs. 22% vs. 48%, respectively) (p<0.001).

## Discussion

The Boston Naming Test (BNT)^9^ is the most frequently used test for visual confrontation naming and might be an important diagnostic tool to differentiate between normal aging and cortical dementia of the Alzheimer type.

A theoretical stage model of normal naming and lexical access can be used.^26,27^

The first stage is perceptual in which the analysis of the characteristics of the object takes place. In the second integrative stage the simultaneous analysis of the primary characteristics is carried out. In the third semantic stage, the visual image has to be matched with semantic knowledge of the object (ordinate, super-ordinate or sub-ordinate). In the fourth lexical stage, the semantic knowledge of an object corresponds to a word (or name) of the object. Finally, in the fifth phonemic stage the actual production of a word takes place.

The qualitative error types made on the BNT can be the result of disruption in one of the stages of the model outlined above. Semantically related errors indicate disruption in the 3^rd^ stage of the model, where integrated information should trigger semantic knowledge. Visual perceptual errors reveal a defect in the first levels, before semantic recognition takes place. Phonological errors reveal a defect in the fifth stage of the model, where phonological processing takes place. A semantic anomia show interruption in the third stage of the model while evocative anomia shows interruption in lexical decision (fourth stage).^26^ Based on this model, aMCI patient have difficulties in the 3^rd^ stage, in which semantic knowledge is processed.

Previous research has found evidence that early in the advance of AD, declines in semantic performance were due to changes in attentional control and/or access processes and that later in the course of AD, declines in performance were best explained as being due to additional breakdowns in the structure of semantic memory.^11^

AD results in a general disruption of the organization and structure of semantic knowledge such that concepts, concept attributes, and links between concepts are lost or degraded because of neural degeneration in critical cortical areas. From this perspective, AD-related declines in picture naming might be attributable to a breakdown in semantic networks responsible for propagating activation to lexical and phonological representations.^11^

Several studies have found no significant difference in total BNT score between aMCI and controls.^13^ Our results show that aMCI patients did not differ significantly in total scores, but showed significantly higher semantic errors compared to controls.

Further longitudinal research should investigate whether aMCI patients with this kind of semantic deficit go on to develop AD or if we can define the aMCI subgroup with mild semantic impairment as a predictor of very early AD. The use of visual confrontation naming as a diagnostic tool for aMCI is important, and further examination should reveal what form intervention in this early stage of AD development can take.
